# Preparation, Characterization and Stability Study of Dutasteride Loaded Nanoemulsion for Treatment of Benign Prostatic Hypertrophy

**Published:** 2014

**Authors:** Mohammad Sajid Ali, Mohammad Sarfaraz Alam, Nawazish Alam, Masoom Raza Siddiqui

**Affiliations:** a *Department of Pharmaceutics, College of Pharmacy, Jazan University, Jazan, K.S.A.*; b *Department of Pharmaceutics, S.B.S College of Pharmacy, Patti, Amritsar, Punjab, India.*; c *Advanced Materials Research Chair, Department of Chemistry, College of Science, King Saud University, Riyadh 11451, Saudi Arabia.*

**Keywords:** Benign prostatic hyperplasia, Dutasteride, Nanoemulsion, Self-life, Stability study, TEM

## Abstract

Benign prostatic hyperplasia (BPH)is the most common condition in aging men, associated with lower urinary tract symptoms. It is caused due to the augmented levels of the androgen dihydrotestosterone. Dutasteride, a 5α-Reductase inhibitor has been recommended for the treatment of BPH upon oral administration. However, long term oral administration of dutasteride may cause sexual problem in man. Therefore the main objective of this study was to develop transdermal patch having nanoemulsion gel of dutasteride in order to enhance physical and chemical stability and eliminate adverse effect of dutasteride. Optimized nanoemulsion was prepared by aqueous phase-titration method and characterized by droplet size, viscosity and refractive index. *In-vitro* skin permeation of dutasteride through rat abdominal skin was determined by the Franz diffusion cell.Significant increase in the steady state flux (*J*_ss_), permeability coefficient (*K*_p_) and enhancement ratio (*E*_r_) was observed in optimized nanoemulsion formulation A1 (*p *< 0.05). The E_r_ of optimized nanoemulsion A1 was found to be 1.52 times with respect to control which indicates transdermal delivery may be better approach for BPH. Stability studies were performed for the period of 3 months. It was found that droplet size, viscosity and refractive index were slightly increased at refrigerator and room temperature in 3 months period. However, the changes in these parameters were not statistically significant (p ≥ 0.05). The shelf-life of optimized nanoemulsion A1 was found to be 2.18 years at room temperature. These results indicated that both physical as well as chemical stability of dutasteride in nanoemulsion formulation.

## Introduction

Benign prostatic hyperplasia (BPH) is the nonmalignant enlargement of the prostate gland resulting due to over-proliferation of the stromal and glandular elements of the prostate (1). It is caused due to the increased levels of the androgen dihydrotestosterone (DHT). In BPH, the microscopic foci within some specific regions of the prostrate grows and forms macroscopic nodule, this macroscopic nodule eventually displaces the normal prostatic tissues which results in the urethral compression. This compression, which is the outcome of the increased cell proliferation and/or impaired apoptosis, causes physical enlargement of the prostate gland and is referred to as static component. In addition, dynamic component comprises sympathetic nerve stimulation causing contraction of prostatic and urethral smooth muscle resulting into outﬂow obstruction (2). The occurrence of BPH is about 70% at the age of 70 years and becomes nearly universal with advancing age. Clinically, BPH causes a constellation of symptoms known as lower urinary tract symptoms (LUTS). The LUTS symptoms include; frequency, hesitancy, urgency, nocturia, slows urinary stream and incomplete emptying (3). 

Earlier treatment of BPH include watchful waiting, transurethral resection of the prostate (TURP) or open prostatectomy. Since the older treatment procedures were of disturbing nature and having potential side effects, many medical therapies have emerged involving the suppression of androgen stimulation of prostatic growth (4). These modern therapies delay or eliminate the obligation of surgery.

5α-Reductase enzyme has emerged as a target for the pharmaceutical treatment of BPH.The treatment is based on the abnormally high activity of the enzyme 5α-Reductase inhumansupshots in excessive DHT levels in peripheral tissues which in turn result in the suppression of androgen action by 5α-reductase inhibitors (5). 

The objective of this study was to develop transdermal patch having nanoemulsion gel of dutasteride (5α-Reductase inhibitor). This drug was selected owing to its successful application in the treatment of BPH. Since the bioavailability of dutasteride is less via oral route, it was aimed at developing transdermal patch. Another reason behind the current study is that in old age it is difficult to take medicine orally (6, 7).

Use of nanoemulsions in transdermal drug deliveryrepresents an important area of research in drug delivery,which enhances the therapeutic efficacy and also thebioavailability of the drugs without any adverse effects.It is also regarded as a promising technique with manyadvantages including, high storage stability, low preparation cost, thermodynamic stability, absence of organicsolvents, and good production feasibility. They have alsomade the plasma concentration profiles and bioavailability of drugs reproducible. These systems are being usedcurrently to provide dermal and surface effects, and for deeper skin penetration. Many studies have shownthat nanoemulsion formulations possess improved trans-dermal and dermal delivery properties *in-vitro* as well as *in-vivo*. Nanoemulsions have improvedtransdermal permeation of many drugs over the conventional topical formulations such as emulsions and gels.

For the development of nanoemulsioneucalyptus oil and oleic acid is used as oil, Tween-20 as surfactant and ethyl alcohol as co-surfactant. Nanoemulsionwas prepared by titration method and different parameters were evaluated.

Nanoemulsions are isotropic, thermodynamically stable, transparent (or translucent) systems of oil, water, surfactant and co-surfactant with a droplet size usually in the range of 20–200 nm (8). Nanoemulsions emerged as a promising tool for the drug delivery owing to its long term stability, ease of preparation and high solubilization of the drug molecules.Recently, there has been a surge in the investigation of nanoemulsions for transdermal delivery(9). 

The aim of this study was to provide an effective screening approach for the proper selection of oils, surfactants, and co-surfactants for the nanoemulsion formulation of steroidal drug which is used in BPH. Dutasteride was selected as a model lipophilic drug for this purpose. These systems often require high surfactant concentration, which may lead to toxicity and irritable problems. Therefore, it is very important to select a suitable surfactant along with its optimum concentration. Determination of the effect of the surfactant to co-surfactant ratio (*S*_mix_) on the nanoemulsion formation region also formed an important consideration of the study.

## Experimental

*Materials*

Dutasteride was obtained as a gift sample from Taj Pharmaceutical *Ltd*. (Mumbai, India) and was used as received. Propylene glycol, monocaprylic ester (sefsol 218) was obtained as a gift sample from Nikko Chemicals (Tokyo, Japan). Diethylenemonoglycol ether (carbitol) and castor oil were purchased from Sigma Aldrich (St. Louis, MO). Isopropyl myristate, glycerol triacetate (triacetin), castor oil, eucalyptus oil, oleic acid were purchased from E-Merck (Mumbai, India). Polyoxyethylenesorbitanmonolaurate (tween-20), polyoxyethylenesorbitanmonostearate (tween-60), polyoxyethylenesorbitanmonooleate (tween-80), ethanol, isopropyl alcohol, PEG 200, propylene glycol, pleurol oleic, brij 35, lecithin were procured from S.D Fine Chemicals (Mumbai, India). Milli Q water was used during the whole experiment. All chemicals and solvents were of analytical grade.

*Screening of excipients*

The most important criterion for screening of components is the solubility of drug in oils, surfactants and co-surfactants.

*Screening of oil*

The solubility of dutasteride in various oils was determined by adding an excess amount of drug in 2 mL of the different oil separately in a 5 mL capacity stopper vials. The content of the vials were mixed using a vortex mixer. The mixture vials were then kept at 25±1.0 °C in an isothermal shaker for 72 h to achieve equilibrium. The equilibrated samples were removed from the shaker and centrifuged at 3,000 rpm for 15 min. The supernatant was taken and filtered through a 0.22-μm membrane filter. Supernatant 10 µL oil was taken and diluted with methanol and concentration of dutasteride was determined in oils using a UV spectrophotometer at 240 nm (10).

*Screening of surfactant and co-surfactant for nanoemulsion*

To find out the suitable surfactant and co-surfactant, the solubility of dutasteride was determined in various surfactants including tween-20, tween -60, tween-80, brij35, lecithin, plurol oleic acid and a combination of two surfactants was taken. The solubility of dutasteride was also checked in co-surfactants such as ethanol, isopropyl alcohol, PEG 200, and polyethylene glycol following the similar procedure as mentioned in oil selection.

*Phase studies*

On the basis of solubility studies, eucalyptus oil: oleic acid (1.5:1) was selected as an oil phase. Tween-20 and ethanol were selected as surfactant and co-surfactant, respectively. Milli Q water was used as an aqueous phase. For the determination of existence zone of nanoemulsion, pseudo ternary phase diagrams were constructed using water titration method (spontaneous emulsification method) (11). Surfactant and co-surfactant (S _mix_) were mixed in different weight ratios (1:1, 2:1, 3:1 and 1:2). These S _mix_ were chosen in increasing concentration of co-surfactant with respect to surfactant. For each phase diagram, oil and specific S _mix_ were mixed well in different ratios. Sixteen different combinations of oil and S _mix_ (1:9, 1:8, 1:7, 1:6, 1:5 1:4, 1:3.5, 1:3, 3:7, 1:2, 4:6, 5:5, 6:4, 7:3, 8:2, and 9:1) were made so that maximum ratio could be covered for the study to delineate the boundaries of the phases formed precisely in the phase diagrams (12). Slow titration with aqueous phase was done for each weight ratio of oil and S_mix_under moderate stirring, and visual observation was used for transparent and easily flowablenanoemulsion. Gels were claimed for those clear and highly viscous mixtures that did not show a change in the meniscus after being tilted to an angle of 90°. The physical state of nanoemulsion was marked on a pseudo three component phase diagram with one axis representing the aqueous phase, second representing oil, and the third representing a mixture of surfactant and co-surfactant at fixed weight ratio (S _mix_ ratio).

*Selection of formulations*

From the pseudoternary phase diagrams showing maximum nanoemulsion area, a number of nanoemulsions with different composition were selected covering the entire range of nanoemulsion occurrence in the phase diagrams with minimum surfactant and maximum water concentration. 0.5 mg dutasteride, which was kept constant in all the selected formulations, was added to the oil phase during the formulation of nanoemulsions. Selected formulations were subjected to various physical stability tests.

*Thermodynamic stability testing of nanoemulsions*

In order to find out the stable nanoemulsion and to discard the unstable or metastable nanoemulsions the placebo nanoemulsions were subjected to following thermodynamic stability studies. 

*Freeze thaw cycle*

Nanoemulsions were kept in deep freezer (at -20 °C) for 24 h. After 24 h the nanoemulsions were removed and kept at room temperature. The thermodynamically stable nanoemulsions returned to their original form within 2-3 min. 2-3 such cycles were repeated.

*Centrifugation studies*

Nanoemulsions after freeze thaw cycle were subjected to centrifugation studies where they were made to undergo centrifugation for 30 min. at 5,000 rpm in a centrifuge. The stable formulations did not show any phase separation or turbidity. 

*Heating cooling cycle*

Six cycles between refrigerator temperature (4 °C) and 40 °C with storage of 48 h were performed. Those formulations which were stable at these temperature, subjected to further study. 

*Characterization of nanoemulsions*

*Globule size analysis*

The droplet size of the nanoemulsions was determined by photon correlation spectroscopy, which analyses the fluctuations in light scattering due to Brownian motion of the particles using a Zetasizer 1000 HS (Malvern Instruments, Worcestershire, UK). Light scattering was monitored at 25 °C at a 90° angle.

*Viscosity*

Viscosity of nanoemulsion was determined by using Brookfield LV rotational viscometer at 2.5, 5, 10 and 20 rpm. Each reading was taken after equilibrium of the sample at the end of two minutes. The samples were repeated three times. The viscosity values at 5 rpm were selected

*Refractive index*

The refractive index of the system was measured by an Abbe refractometer (Bausch and Lomb Optical Company, Rochester, NY) by placing one drop of the formulation on the slide in triplicate at 25 °C.

*pH Measurements*

The apparent pH of the formulations was measured by a pH meter (Mettler Toledo MP 220, Greifensee, Switzerland) in triplicate at 25 °C.

*Transmission Electron Microscopy (TEM)*

Morphology and structure of the nanoemulsion were studied using Morgagni 268D electron microscope (Fei Company, Netherlands) operating at 70 kV capable of point-to-point resolution. Combination of bright field imaging at increasing magnification and of diffraction modes was used to reveal the form and size of the nanoemulsion. In order to perform transmission electron microscopy (TEM) observations, a drop of the nanoemulsion was suitably diluted with water and applied on a carbon-coated grid, then treated with a drop of 2% phosphotungstic acid and left for 30 s. The coated grid was dried and then taken on a slide and covered with a cover slip and observed under the microscope.

*Hydrogel thickened nanoemulsion*

The very low viscosity often exhibited by nanoemulsion is not suitable for transdermal use. The viscosity can be increased by adding thickening agents, which also change the appearance of the system, usually influencing drug release. Recently, the gel matrices such as carbopol 934, sodium alginate, ethyl cellulose, and HPMC have been used to prepare the nanoemulsion based gel for improving the viscosity of nanoemulsion(13, 14). The selection of polymer for preparing gel is normally based on the character of external phase (oil for w/o type and water for o/w type). Because dutasteridenanoemulsion is a type of o/w type, so carbopol 934 was selected for preparation of nanoemulsion gel. For preparation of nanoemulsion gel 1% carbopol 934 dispersed in sufficient quantity of distilled water. This dispersion was kept in dark for 24 h for complete swelling of carbopol 934. Prepared nanoemulsion was added slowly to carbopol 934 dispersion. 0.5% w/w of triethanolamine (TEA) was added in this mixture to neutralize carbopol 934. Then by mixing hydrogel thickened nanoemulsion was obtained.

*In-vitro skin permeation studies*

The protocol to carry out *in-vitro* permeation studies was approved by the Institutional Animal Ethics Committee, S.B.S College of Pharmacy, Patti, Amritsar, Punjab, India. The committee's guidelines were followed for the studies. *In-vitro* skin permeation studies were performed on a fabricated Franz diffusion cell with an effective diffusional area of 5.24 cm^2^ and 5 mL of receiver chamber capacity using rat abdominal skin. The full-thickness rat skin was excised from the abdominal region, and hair was removed with an electric clipper. The subcutaneous tissue was removed surgically, and the dermis side was wiped with isopropyl alcohol to remove adhering fat. The cleaned skin was washed with distilled water and stored in the deep freezer at -21 °C until further use. The skin was brought to room temperature and mounted between the donor and receiver compartment of the Franz diffusion cell, where the stratum corneum side faced the donor compartment and the dermal side faced the receiver compartment. Initially, the donor compartment was empty and the receiver chamber was filled with phosphate buffer (pH 7.4). The receiver fluid was stirred with a magnetic rotor at a speed of 100 rpm, and the assembled apparatus was placed in the oven and the temperature was maintained at 37 ± 1 °C. All the receiver fluid was replaced every 30 min to stabilize the skin. It was found that the receiver fluid showed negligible absorbance after 4.5 h and beyond indicating complete stabilization of the skin. After complete stabilization of the skin, 1 mL of nanoemulsion formulation (0.5 mg/mL dutasteride) was placed into each donor compartment and sealed with paraffin film to provide occlusive conditions. Samples were withdrawn at regular intervals (0.5, 1, 2, 3, 4, 5, 6, 8, 10, 12, and 24 h), filtered through a 0.45 membrane filter, and analyzed for drug content by UV spectrophotometer at λ_max_ of 240 nm (10).

*Permeation and distribution data analysis*

The cumulative amount of dutasteride permeated through the albino rat skin (Q, μg/cm^2^) was plotted as a function of time (t, h) for optimized nanoemulsion formulation A1, nanoemulsion gel of A1 and control. Control group represents 30% of S_mix_ (1:1) Tweeen-20 and Ethanol, solution containing 0.5 mg in mL of dutasteride without oil mixture.The permeation rate (flux) at the steady state (J_ss_ ,μg/cm^2^ /h) and lag time were calculated from the slope and intercept of the straight line obtained by plotting the cumulative amount of dutasteride permeated per unit area of skin versus time at steady-state condition, respectively. Permeability coefficient (K _p_) was calculated by dividing the flux by initial drug concentration (C_0_) in the donor portion of cell as given below (15):

*K *_p_ *= J*_ss_ */C*_0_

Enhancement ratio (E_r_) was calculated by dividing the J_ss_ of the respective formulation by the J_ss_ of the control formulation as given below:

*E *_r_ *= J*_ss_ *of formulation/J*_ss_ *of control*

*Histopathology studies*

Abdominal skin of Wistar rats was treated with the optimized dutasteridenanoemulsion gel of A1. After 24 h, the rats were killed and skin samples were taken from untreated (control) and treated areas. Each specimen was stored in 10% formalin solution in phosphate buffer saline (pH 7.4). The specimens were cut into sections vertically. Each section was dehydrated using ethanol embedded in paraffin wax for fixing and stained with hematoxylin and eosin. These samples were then observed under light microscope (Motic, Japan) and compared with control samples.

*Stability studies as per ICH guidelines*

Stability studies on optimized nanoemulsion were performed by keeping the sample at refrigerator temperature (4 °C) and room temperature (25 °C). These studies were performed for the period of 3 months. The droplet size, viscosity and refractive index were determined at 0, 1, 2 and 3 months. Accelerated stability studies were also performed on optimized nanoemulsion as per international conference on harmonization (ICH) guidelines. Three batches of optimized formulation were taken in glass vials and were kept at accelerated temperature of 30, 40, 50 and 60 °C at ambient humidity. The samples were withdrawn at regular intervals of 0, 1, 2 and 3 months. These samples were analyzed for drug content by stability-indicating HPLC method at a wavelength of 241 nm(16). The chromatographic column used was a reverse phase 25 cm X 4.6 mm, *i.d*., 5 im, C18 DB reversed phase column (Phenomenox). The mobile phase was methanol: water (90:10) with the flow rate of 1.25 mL/min. The retention time (Rt) of drug was 5.24 min. Zero time samples were used as controls (100% drug). Analysis was carried out at each time interval by taking 100 µL of each formulation and diluting it to 5 mL with methanol and injecting into the HPLC system at 241 nm. The solubility of sample in methanol was 63.8 mg/mL. In addition, samples of pure oil (combination of eucalyptus oil and oleic acid), pure surfactant and co-surfactant (S) were run separately to check interference of the excipients used in the formulations. 

The amount of drug decomposed and the amount remaining (undecomposed drug) at each time interval was calculated. Order of degradation was determined by the graphical method (17). Degradation rate constant (K) was determined at each temperature. Arrhenius plot was constructed between log K and 1/T to determine the shelf-life of optimized nanoemulsion formulation. The degradation rate constant at 25 °C (K_25_) was determined by extrapolating the value of 25 °C from Arrhenius plot. The shelf-life (T_0.9_) for each formulation was determined by using the formula: 

 T_0.9_ = 0.1054/ K_25_

## Result and Discussion

The most important criteria for selection of all the nanoemulsion components is that all the excipients should be pharmaceutically acceptable for transdermal application, depending upon the requirement and falling under the generally-regarded-as-safe category.

*Screening criteria for oil selection*

Lipophilic drugs are preferably solubilized in o/w nanoemulsions, whereas w/o systems seems to be a better choice for hydrophilic drugs. Drug loading per formulation is a very critical design factor in the development of nanoemulsion systems for poorly soluble drugs, which is dependent on the drug solubility in variety of formulation components. The volume of the formulation should be reduced as much as possible to deliver the therapeutic dose of the drug in an encapsulated form. Solubility of the drug in the oil phase is an important criterion for the selection of oils. This factor is important in the case of transdermal formulation development, as the capacity of nanoemulsion to maintain the drug in solubilized form is greatly influenced by the solubility of the drug in the oil phase (18). 

**Figure 1:**
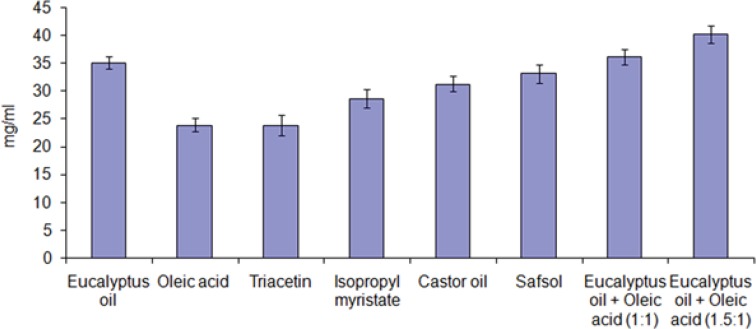
Solubility of dutasteride in different oils at 25 °C (mean ± SD, n = 3).

Figure 1 depicts the solubility of dutasteride in different oils. The solubility of dutasteride was found to be highest in eucalyptus oil: oleic acid (1.5:1) 40.24 ± 1.56 mg/mL as compared to other oils. This may be attributed to the polarity of the poorly soluble drugs that favors their solubilization in small/medium molar volume of oils, such as medium-chain triglycerides or mono- or diglycerides. Oleic acid is used as penetration enhancer as well as medium for drug dissolving (19). Eucalyptus oil also act as medium for drug solubilization as well as penetration enhancer and is frequently used as an oil phase in steroidal drug (20). Low drug solubility would require incorporation of more oil to incorporate the target drug dose, which in turn would require higher surfactant concentration to achieve oil solubilization, which might increase the toxicity of the system. Thus, eucalyptus oil: oleic acid (1.5:1) was selected as the oil phase for the development of nanoemulsion formulation.

*Screening criteria for surfactants*

One of the most critical problems associated to the nanoemulsion-based systems is the toxicity of the components. Large amounts of surfactants may cause skin irritation, when administered topically. So the proper selection of surfactants becomes essential. Thus, it is importantto determine the surfactant concentration properly and use it in minimum concentration in the formulation. Nonionic surfactants are comparatively less toxic than their ionic counterparts and normally have lower CMCs. Also, o/w nanoemulsion dosage forms for transdermal use based on nonionic surfactants offers excellent *in-vivo *stability(21, 22). Therefore, it is very crucial to select proper surfactants. Another important criterion in the selection of surfactant is HLB value. Hydrophilic surfactant and co-surfactant are considered to prefer the interface and lower the necessary energy to form the nanoemulsions which in turn improves the stability. For example, the required HLB value to form o/w nanoemulsion is greater than 10. The right blend of low and high HLB surfactants leads to the formation of a stable nanoemulsion upon dilution with water.

**Figure 2:**
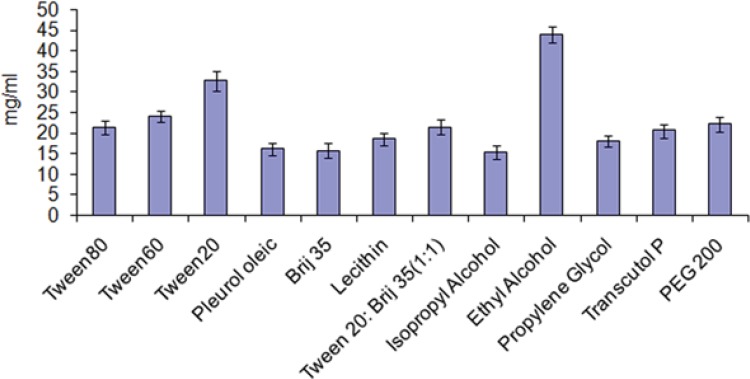
Solubility of dutasteride in different surfactants and co-surfactants at 25 °C (mean ± SD, n = 3).

After finalizingeucalyptus oil: oleic acid (1.5:1) as oil phase, the main aim remains is to identify the surfactant that has the highest solubilization capacity for the oil. In the present study, five surfactants including lecithin, brij35, tween-20, tween-60 and tween-80 were selected for screening. Nonionic surfactants were selected because of they are known to be less affected by pH and changes in ionic strength. They are also regarded as safe, and biocompatible. During the study ionic surfactants were omittedbecause of their toxicological concerns. The selection of the surfactant was based on solubility of drug without addition of co-surfactant. It was observed that tween-20 has greatest solubility for the drug (Figure 2).

*Screening of co-surfactants*

Short to medium-chain alcohols (C3-C8) are commonly added as co-surfactants to reduce the interfacial tension and increase the fluidity of the interface (23). They also increase the mobility of the hydrocarbon tail and allow greater penetration of the oil into this region. Alcohols may also increase the miscibility of the aqueous and oily phases due to its partitioning between these phases. Therefore, ethanol, isopropyl alcohol, 1-butanol, and propylene glycol were selected as co-surfactants. Another reason for the selection of ethanol is its maximum solubility for the drug (Figure 2) and increased permeation when incorporated into formulations and are relatively safe. All the co-surfactants studied are the pharmaceutically acceptable ingredients.

*Preparation of pseudo ternary phase diagram*

**Figure 3:**
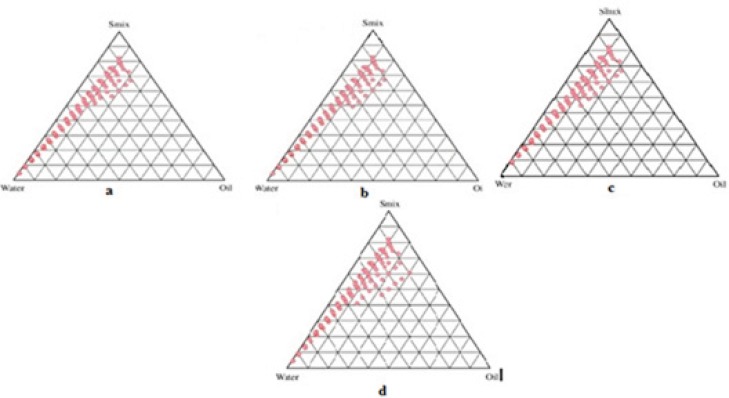
Pseudoternary phase diagrams indicating nanoemulsion region at different S_mix_ ratios: a (S_mix_1:1), b (S_mix_2:1), c (S_mix_ 3:1), d (S_mix_ 1:2).

Formulations were carefully observed so that the metastable systems are not selected.Although the free energy required to form a nanoemulsion is very low yet the formation is thermodynamically spontaneous (24). Effect of surfactant and co-surfactant mass ratio on nanoemulsion formation was evaluated for the further optimization of the system. Low nanoemulsion area was observed when tween-20 was used alone without co-surfactant, *i.e*., at the *S*_mix_ ratio 1:0. Possibly, when the co-surfactant is absent or present at lower concentrations, the surfactant is not able to sufficiently reduce the o/w interfacial tension. An o/w nanoemulsion region was found towards the water-rich apex of the phase diagram. The maximum concentration of oil that could be solubilized can be seen in the phase diagram. When co-surfactant was added with surfactant in equal amounts, a higher nanoemulsion region was observed, perhaps because of the further reduction of the interfacial tension and increased fluidity of the interface at *S*_mix_ 1:1 (Figure 3a). On further increasing the surfactant concentration *i.e*., at *S*_mix_ 2:1 (Figure 3b), the nanoemulsion region increased in size as compared to the region in *S*_mix_ 1:1. When the surfactant concentration is further increased in the *S*_mix_ ratio of 3:1 (Figure 3c), a decrease in the nanoemulsion region was observed when compared with *S*_mix_ 2:1. It can be assumed that, when surfactant concentration was increased in comparison to co-surfactant, the nanoemulsion region increased up to the 2:1 *S*_mix_ ratio, but in the 3:1 ratio, it was decreased, indicating that the optimum emulsification has been achieved. Therefore, there was no need to attempt a *S*_mix_ ratio of 4:1. Therefore, the areas of one phase nanoemulsion zones are dependent on surfactant composition (25). When the co-surfactant concentration with respect to surfactant was increased to the *S*_mix_ 1:2 (Figure 3d) it was observed that the nanoemulsion area decreased as compared to *S*_mix_ 1:1. When co-surfactant concentration was further increased to make *S*_mix_ 1:3, a further decrease in the area was achieved. A narrower nanoemulsion field at *S*_mix_ 1:2 and *S*_mix_ 1:3 were most likely due to a decrease in surfactant concentration by the increased presence of ethyl alcohol. It could be observed that the formulations prepared from phase diagrams in which the nanoemulsion area was extended towards an aqueous-rich apex might be diluted to a larger extent.

The surfactant and co-surfactant mass ratio had been found to be a key feature to influence the phase properties *i.e*. size and position of nanoemulsion region (26, 27). The type and concentration of oil employed, also plays a role (28, 29). *S*_mix_ 2:1 showed the maximum area as compared to the other ratios. Such an effect was attributed to differences in the packing of surfactant and co-surfactant at the o/w interface. It was also observed that decreasing the oil level led to an increase in the area of nanoemulsion formation. This fact suggested that the oil constitutes the inner phase of the nanoemulsion droplets, which is consistent with a direct o/w-type structure. The customary preference is to select formulations with the lowest surfactant concentration for transdermal administration. This is usually not obtained with formulations that contain the highest amount of surfactant since high surfactant concentration decreases the thermodynamic activity of the drug in the vehicle, and the affinity of the drug to the vehicle becomes greater (30). Therefore, formulations should be selected having low concentration of *S*_mix._

*Thermodynamic stability tests of drug loaded nanoemulsion*

In order to eliminate the possibility of metastable formulations, stress testing is vital. Some symbolic formulations were taken from the o/w nanoemulsion region of the phase diagram constructed at different *S*_mix_ , and were subjected to the thermodynamic stability tests such as heating cooling cycle, freeze thaw cycle, and centrifugation. Results of thermodynamically stable formulations were shown in (Table 1). Thermodynamic stability confers long shelf life to the nanoemulsion as compared to ordinary emulsions. It differentiates them from emulsions that have kinetic stability and will eventually phase-separate. Thermodynamically stable formulations were selected for further studies.

**Table 1:** Composition of selected nanoemulsion formulations

**Oil used: Eculaptus oil: Oleic acid (1.5:1) , Surfactant used: Tween-20,** **Cosurfactant used: Ethanol, External phase: Distilled water**									
**mix** **Ratio**	**Formulation** **code**	**Oil** **(%)**	**S** **mix** **(%)**	**Water** **(%)**	**H/C**	**Cent**	**Freeze**	**Result**	**Dutasteride** **(in 1 mL formulation)**
1:1	A1	5	30	65	√	√	√	Passed	0.5 mg
	A2	10	33	57	X	√	√	Failed	0.5 mg
	A3	15	40	45	√	√	√	Passed	0.5 mg
2:1	B1	5	36	54	√	√	√	Passed	0.5 mg
	B2	10	40	50	√	√	X	Failed	0.5 mg
	B3	15	35	50	√	X	√	Failed	0.5 mg
3:1	C1	5	31	64	√	√	√	Passed	0.5 mg
	C2	10	38	52	√	√	X	Failed	0.5 mg
	C3	15	44	41	√	√	√	Passed	0.5 mg
1:2	D1	5	33	62	X	√	√	Failed	0.5 mg
	D2	10	45	45	√	√	√	Passed	0.5 mg
	D3	15	50	35	√	√	X	Failed	0.5 mg

H/C = Heating and cooling cycle, Cent= Centrifuge

*Characterization of the selected nanoemulsions*

The nanoemulsions which qualified the stress testing were further selected for characterization process.

*Globule size analysis*

From Table 2, it is evident that the droplet size increased with the increase in concentration of the oil in the formulations. It was also observed that the droplet size of all the formulations was in the nano range. The low polydispersibility values observed for all the formulations indicated uniformity of droplet size within each formulation.

**Table 2:** The Characteristics of the nanoemulsion formulations

Formulation Code	Mean Globule Size (nm)	Poly dispersivity	Viscosity (mP)	pH	Refractive Index
A1	58.83 ± 0.73	0.177 ± 0.02	38.45±1.62	5.8±0.03	1.431±0.023
A3	85.25 ± 0.42	0.194 ± 0.04	41.53±1.37	5.6±0.05	1.463±0.017
B1	60.74 ± 0.83	0.191 ± 0.03	39.39±1.54	5.7±0.03	1.438±0.022
C1	69.66 ± 0.73	0.196 ± 0.07	39.85±1.42	5.5±0.03	1.439±0.015
C3	88.74 ± 0.93	0.201 ± 0.06	42.81±1.38	5.5±0.04	1.461±0.018
D2	65.74 ± 0.31	0.199 ± 0.02	39.55±1.64	5.7±0.05	1.441±0.016

Value represents as mean±SD (n=3)

**Figure 4:**
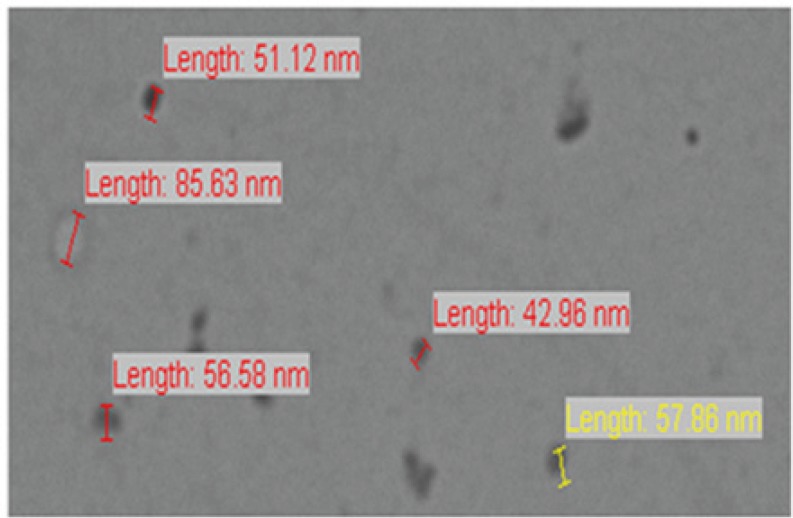
TEM image of optimized nanoemulsion A1

**Figure 5:**
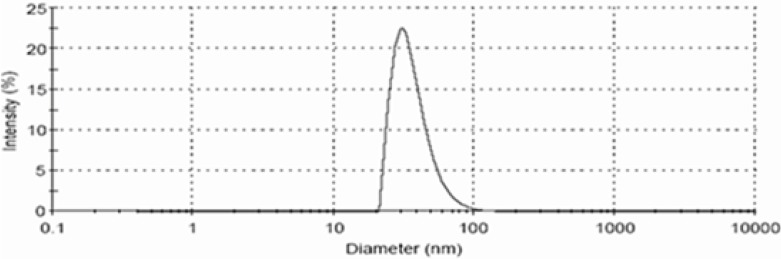
Droplet size and size distribution of optimized nanoemulsion A1

The droplets in the nanoemulsion appear dark, and the surroundings are bright; a “positive” image was seen using TEM (Figure 4). Some droplet sizes were measured using TEM, as it is capable of point-to-point resolution. The uniformity of particles size was investigated with the help of size distribution analysis as shown in (Figure 5).

*Viscosity*

The viscosity study revealed that viscosity tends to increase with increase in the oil content. As the oil content was increased from 5% w/w to 20% w/w, an increase in the viscosity of the formulations was observed, the results are mentioned in Table 2. The viscosity of formulation A1 was significantly lower than that of the other formulations (*p*<0.05), which might be due to its lower oil content. Overall, very low viscosity of the formulations was observed, which is expected for nanoemulsions.

*pH Measurements*

The apparent pH of all formulations were measured by pH meter in triplicate at 25 ± 1 °C and found to in between 5-6 (Table 2). This pH range is optimum for transdermal formulation. 

*Refractive index*

Refractive index is the net value of the components of nanoemulsion and indicates the isotropic nature of the formulation. The mean value of the refractive index for all the formulations was given in (Table 2). The lowest values of RI was seen in A1 formulation, might be due to a increase in water content, as water has a comparatively lower refractive index (the refractive index of water is 1.334).

*In-vitro skin permeation studies*

**Figure 6:**
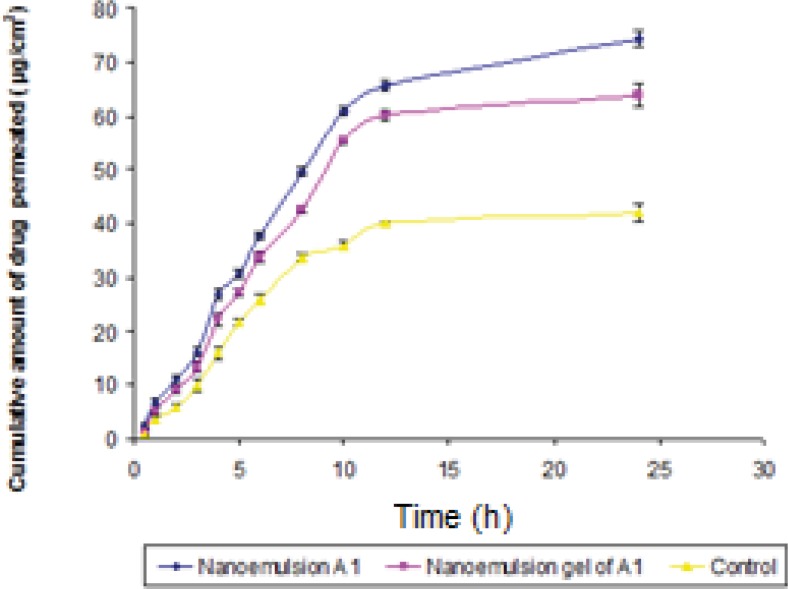
Comparison of *in-vitro* skin permeation of dutasteride from optimized nanoemulsion A1, nanoemulsion gel and control in phosphate buffer (pH 7.4).

The permeation ability of dutasteride loaded nanoemulsion; nanoemulsion gel and control were evaluated using the *in-vitro* permeation experiments. A steady increase of dutasteride in the receptor chamber with time was observed. The permeation profiles of nanoemulsions were in accordance with the Fick's diffusion equation. On the basis of permeation studies, it was found that the formulation A1 consisting of 5% oil phase, 30% (S_mix 1:1_ ) and 65% distilled water exhibited highest cumulative amount of drug permeated (74.36 μg/cm ^2^) with the flux of 3.09 (μg/cm ^2^ /h) after 24 h. Permeability coefficient (K_p_ ) was found to be 6.19 x 10^-3^ cm/h. The cumulative amount of drug permeated from nanoemulsion gel of A1 was found to be 63.92 μg/cm^2^ (Figure 6). There was statically significant decrease (P ≤ 0.05) in cumulative amount of drug permeated in 24 h from optimized gel of A1 in comparison to nanoemulsion A1. Gel was formulated in view of ease in fabrication of transdermal patch. The E_r_ of nanoemulsion gel was found to be 1.52 times with respect to control.

*Histopathology studies*

**Figure 7:**
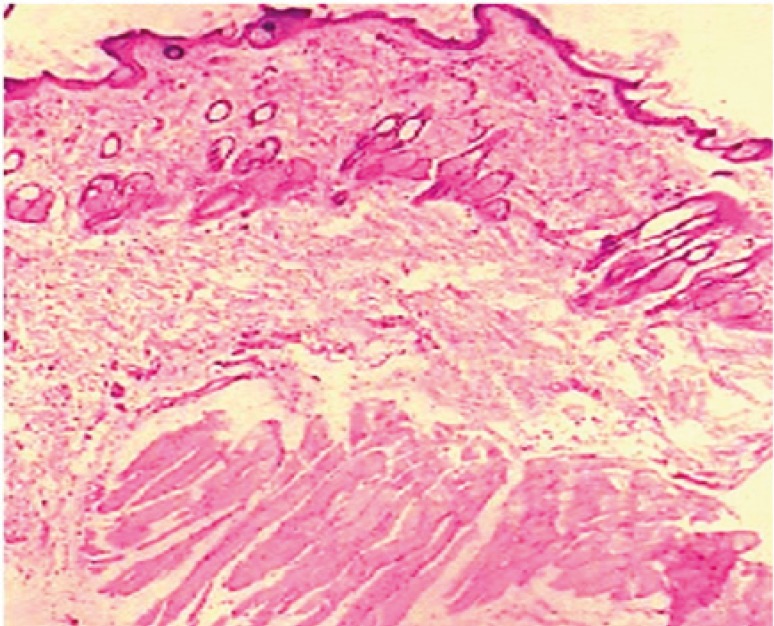
Light power photomicrograph of control skin

**Figure 8:**
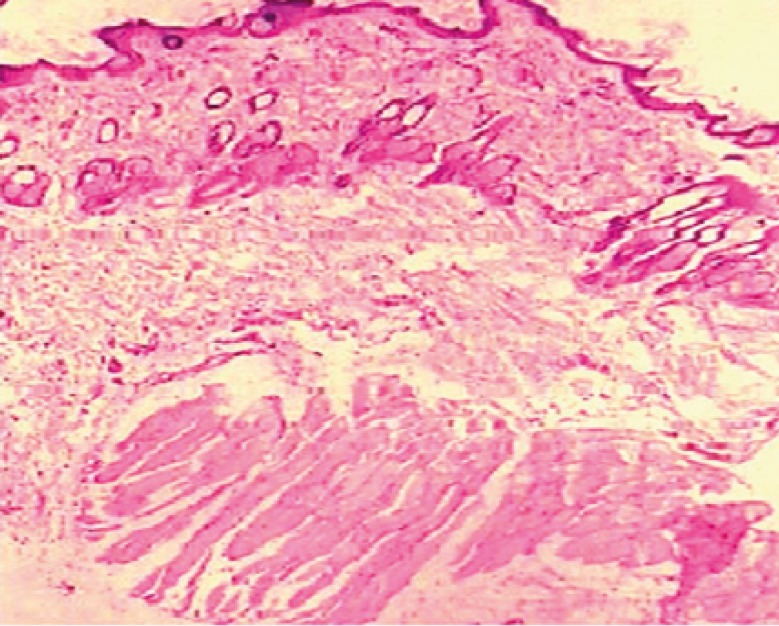
Light power photomicrograph of treated skin

The influence of dutasteride loaded nanoemulsion gel on anatomical structure of the rat skin was assessed with the help of histopathological studies. After observation of light power photomicrograph of control and treated skin (Figure 7) and (Figure 8) it was found that no significant difference was seen in the shape and size of the tissue of rat skin.

*Stability studies*

The purpose of stability testing is to produce proof that how the quality of a drug substance or drug product varies with time under the influence of a multiple environmental factors, such as temperature, humidity, and light, and to establish a re-test period for the drug substance or a shelf-life for the drug product and recommended storage conditions. An ideal drug product must be fully characterized physically, chemically and microbiologically at the start of study and throughout the intended shelf-life period. Therefore, optimized nanoemulsion formulation was characterized for droplet size, viscosity and RI for the period of three months. During stability studies droplet size, viscosity and RI were determined at 4 °C and 25 °C. These parameters were determined at 0, 1, 2 and 3 months. It was found that droplet size, viscosity and RI were slightly increased in time at both temperatures (Table 3). These parameters were compared for statistical significance by one-way analysis of variance (ANOVA) followed by Tukey-Kramer multiple comparisons test using Graph Pad Instat software (Graph Pad Software Inc., CA, USA). The changes in these parameters were not statistically significant (P ≥ 0.05). These results indicated that optimized formulation is stable as there were no significant changes in physical parameters (droplet size, viscosity and RI). For accelerated stability studies, samples were withdrawn at regular intervals of 0, 1, 2, and 3 months. The samples were analyzed for their drug content by high performance liquid chromatography (HPLC) analysis at a wavelength of 241 nm. The degradation of dutasteride was very slow at each temperature which indicated the chemical stability of dutasteride in the nanoemulsion formulation. The optimized nanoemulsion was found to be stable chemically as well as physically, it was concluded that it is suitable for transdermal delivery. 

**Table 3:** Droplet size, viscosity and RI of optimized nanoemulsion A1 during storage

Time in (months)	Temperature (°C)	Mean droplet size (nm) ± SD (n=3)	Mean Viscosity (mP) ± SD (n=3)	RI ± SD (n=3)
0	4.0 ± 0.5	48.12 ± 1.83		1.431±0.023
1	4.0 ± 0.5	58.95 ± 1.76	38.51 ± 1.71	1.433±0.021
2	4.0 ± 0.5	59.12 ± 1.83	38.94 ± 1.53	1.434±0.024
3	4.0 ± 0.5	48.89 ± 1.89	38.98 ± 1.61	1.436±0.027
0	25 ± 0.5	59.12 ± 1.83	38.45 ± 1.62	1.431±0.023
1	25 ± 0.5	58.96 ± 1.93	38.71 ± 1.71	1.432±0.028
2	25 ± 0.5	60.33 ± 2.31	38.83 ± 1.39	1.437±0.025
3	25 ± 0.5	60.71 ± 2.47	39.19 ± 1.37	1.439±0.021

**Figure 9:**
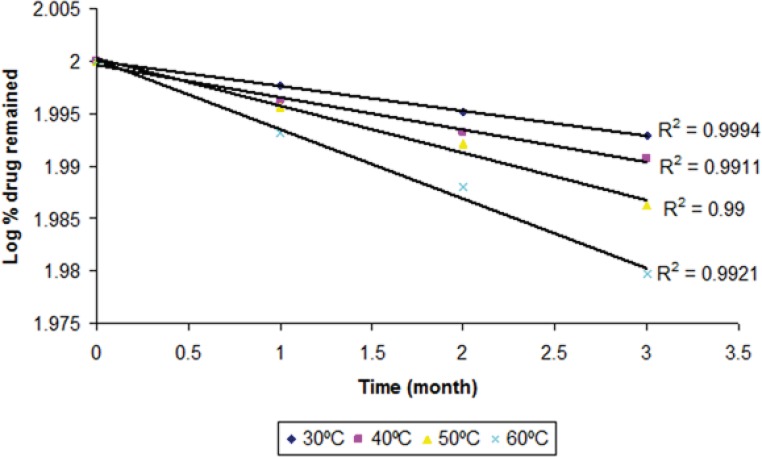
First order degradation kinetics of dutasteride from optimized nanoemulsion A1 at different temperatures

**Figure 10:**
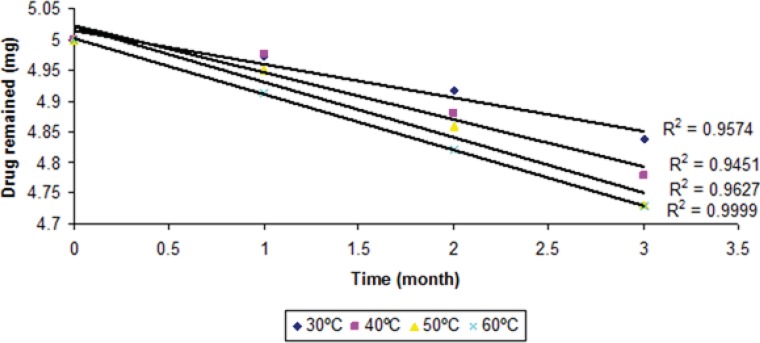
Zero order degradation kinetics of dutasteride from optimized nanoemulsion A1 at different temperatures.

The degraded and remained concentration of dutasteride at different temperatures is shown in (Table 4). The order of degradation was determined by graphical method at each temperature. The order of degradation was found to be first order (Figure 9). In first order degradation, the rate of degradation is independent of the concentration of reacting species. However, the rate of degradation is directly proportional to the first power of the concentration of a single reactant in first order degradation. The correlation coefficients of first order degradation were significant as compared to correlation coefficients of zero order degradation. Log % remained at each temperature as shown in (Figure 9) and (Figure 10) (p < 0.05). 

**Table 4:** Degradation of optimized nanoemulsion A1

**Time** **(Days)**	**Temp ** **(ºC)**	**Drug** **content** **(mg)**	**Drug content degraded (mg)**	**%drug remaining**	**Log % drug remaining**
0	30 ± 0.5	5	0	100	2
30	30 ± 0.5	4.972	0.028	99.448	1.9976
60	30 ± 0.5	4.917	0.083	98.87	1.9951
90	30 ± 0.5	4.838	0.162	98.378	1.9929
0	40 ± 0.5	5	0	100	2
30	40 ± 0.5	4.975	0.025	99.128	1.9962
60	40 ± 0.5	4.880	0.12	98.446	1.9932
90	40 ± 0.5	4.778	0.222	97.881	1.9907
0	50 ± 0.5	5	0	100	2
30	50 ± 0.5	4.95	0.05	99.991	1.9956
60	50 ± 0.5	4.86	0.14	98.197	1.9921
90	50 ± 0.5	4.731	0.269	96.894	1.9863
0	60 ± 0.5	5	0	100	2
30	60 ± 0.5	4.912	0.088	98.423	1.9931
60	60 ± 0.5	4.821	0.179	97.274	1.988
90	60 ± 0.5	4.729	0.271	95.433	1.9797

Therefore for first order degradation, Log % of drug remaining was plotted against time (Figure 9) and K was calculated from the slope of the curve at each temperature.

The values of K at each temperature are given in the (Table 5). The log of drug remaining was plotted against time (months). Slope of each line was obtained and K was calculated by the formula:

Slope= –K/2.303

Where, K is the degradation rate constant.

**Figure 11:**
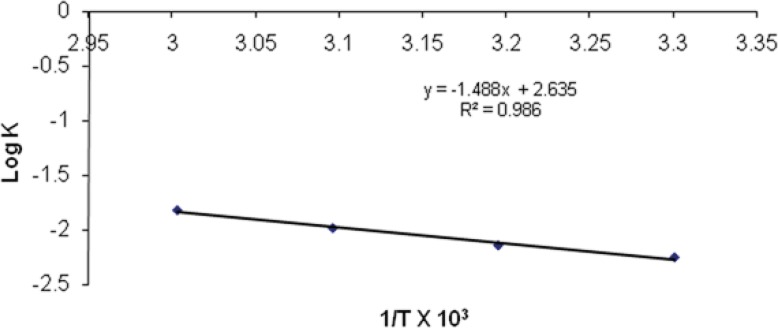
Arrhenius plot between Log K and 1/T for optimized nanoemulsion A1.

The effect of temperature on the degradation was studied by plotting log K v/s 1/T. (Figure 11). The value of K at 25°C (K_25_) was obtained by extrapolation of the plot and shelf-life was then calculated. The shelf-life of optimized nanoemulsion formulation was found to be 2.18 years. 

**Table 5:** Observation table for calculation of shelf-life of nanoemulsion A1

**Temperature** **(ºC)**	**Slope**	**K×10** ^-3^ **(month** ^-1^ **)**	**Log K**	**Absolute Temperature (K)**	**1/T × 10** ^3^
30	-0.0024	5.527	-2.25749	303	3.30033
40	-0.0031	7.139	-2.14634	313	3.19488
50	-0.0045	10.364	-1.98449	323	3.09597
60	-0.0066	15.201	-1.81816	333	3.00300
25		4.017	-2.39579	298	3.35570

## Conclusion

For the preparation of nanoemulsion titration method was used. The proposed method is very simple and by this method nanoemulsions can be prepared without involving any sophisticated equipment. Only critical step is of proper selection of components for an efficient nanoemulsion formulation. Low surfactant containing nanoemulsion was taken for further studyso as to decrease or eliminate the toxicity or irritation of the nanoemulsion formulations. In the present study oleic acid is used as oil phase and has good penetration ability through the skin for transdermal preparation. The E_r_ of optimized formulation A1 was found to be 1.52 times with respect to control. Stability study was performed as per ICH guide line and optimized formulation was found to be stable. The droplet size, viscosity and RI of optimized nanoemulsion formulation shows that there was not significantly changed during 3 months of storage suggesting that prepared nanoemulsion was physically stable. The degradation of dutasteride after 3 months of storage was also slowest in the formulation. Slower degradation of dutasteride indicated the chemical stability of dutasteride in nanoemulsion. The shelf-life of nanoemulsion formulation was found to be 2.18 years at room temperature. These results indicated that both physical as well as chemical stability of dutasteride.
